# A database of life-history traits of European amphibians

**DOI:** 10.3897/BDJ.2.e4123

**Published:** 2014-10-30

**Authors:** Audrey Trochet, Sylvain Moulherat, Olivier Calvez, Virginie M Stevens, Jean Clobert, Dirk S Schmeller

**Affiliations:** † Université Toulouse 3 Paul Sabatier, CNRS, ENFA, UMR5174 EDB (Laboratoire Évolution & Diversité Biologique), Toulouse, France; ‡ Station d'Ecologie Expérimentale du CNRS at Moulis, Moulis, France; § TerrOïko, Revel, France; | Helmholtz-Centre for Environmental Research - UFZ, Department of Conservation Biology, Leipzig, Germany; ¶ Université de Toulouse; UPS, INPT; Laboratoire Ecologie Fonctionnelle et Environnement (Ecolab); CNRS, Toulouse, France

**Keywords:** Amphibians, life history traits, Europe

## Abstract

In the current context of climate change and landscape fragmentation, efficient conservation strategies require the explicit consideration of life history traits. This is particularly true for amphibians, which are highly threatened worldwide, composed by more than 7400 species, which is constitute one of the most species-rich vertebrate groups. The collection of information on life history traits is difficult due to the ecology of species and remoteness of their habitats. It is therefore not surprising that our knowledge is limited, and missing information on certain life history traits are common for in this species group. We compiled data on amphibian life history traits from literature in an extensive database with morphological and behavioral traits, habitat preferences and movement abilities for 86 European amphibian species (50 Anuran and 36 Urodela species). When it were available, we reported data for males, females, juveniles and tadpoles. Our database may serve as an important starting point for further analyses regarding amphibian conservation.

## Introduction

Amphibians are ectotherms, and all aspects of their life history are strongly influenced by the external environment, including weather and climate. Amphibians are currently the most threatened taxonomic group worldwide (Temple and Cox 2009, IUCN 2011). The major threats acting on amphibian populations are habitat loss and habitat fragmentation, pollution, global change or disease exposure (Beebee and Griffith 2005, Blaustein and Kiesecker 2002, Houlahan et al. 2000, Stuart et al. 2004). Habitat fragmentation is actually recognized as the major treat of amphibian decline (Chanson et al. 2008, Marsh and Trenham 2001) by its strong impact on population functioning, in particular in amphibians. Indeed, habitat fragmentation can decrease the size of habitat patches, and also the distances between habitat patches (Fahrig 2003, Chanson et al. 2008, Reh and Seitz 1990). Consequently, this loss of connectivity should negatively affect population functioning, by limiting dispersal events between patches and by increasing inbreeding risk (Sjögren-Gulve 1994).

The impact of global warming on amphibian populations has been reported several times (Beebee 2002, Blaustein et al. 2001, Blaustein and Kiesecker 2002, Corn 2003, Houlahan et al. 2000, Araujo et al. 2006, Alford et al. 2006). For example, the breeding phenology of anurans adapted to breeding in early spring might be shifted to even earlier breeding in recent years in response to warmer spring temperatures (Beebee 1995, Parmesan 2006, Klaus and Lougheed 2013). These responses may not be universal among amphibians and remain a matter of debate (Beebee 2002, Corn 2003, Reading 1998). It is undoubted that rising temperatures, changes in precipitation and UV-radiation are considered stressful and might be associated with disease outbreaks in amphibian populations (Blaustein and Kiesecker 2002, Kiesecker and Blaustein 1995, Walker et al. 2010). UV-B may, however, also enhance tadpole growth in some species (Pahkala et al. 2003), but with not yet anticipated effects on survival of metamorphs and population dynamics.

Our database on life history traits of 86 European Amphibian species is an important prerequisite for understanding change in amphibian life history, community composition and migration behavior. Such data is important to inform the Essential Biodiversity Variables (Pereira et al. 2013), develop new indicators and ultimately inform the decision-making process to improve amphibian conservation.

## General description

### Purpose

**Figure 1.**
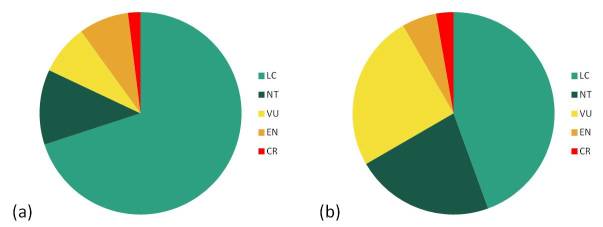
Proportion of (a) Anura (N=50) and (b) Urodela species (N=36) within IUCN categories used in our database. Data were extracted in 2013 from information found on the IUCN website (IUCN 2011). IUCN categories: LC = least concern, NT = near threatened, VU = vulnerable, EN = endangered, CR = critically endangered.

Our database summarizes life history traits, including morphology, reproductive strategies, movement abilities, habitat preferences, distribution and IUCN status for all European amphibian species (N=86), all in all 44 traits subdivided into 253 modalities. Our database comprises information from 304 scientific publications assembled by searching Web of Science®, Amphibiaweb (AmphibiaWeb 2012) and herpetological books. The IUCN status, from 1 (least concern) to 6 (extinct in the wild), and their population trends (-1: decreasing, 0: stable or +1: increasing) were extracted from the IUCN red list website (IUCN 2011). In total, we were able to compile data for 50 Anurans and 36 Urodela (Fig. 1). When several values were available for a continuous trait, we averaged them across studies (i.e. between populations). When they are available, data for males, females, juveniles (larvae) and tadpoles were reported. Summary of mean data, range and missing values are given in Table 2.

Because habitat fragmentation is currently the most threat affecting amphibian populations, movement specific data could help for conservation plans. By this way, we first selected traits related to movement abilities. Then, the costs associated with movements, and particularly with dispersal, might constrain the allocation of resources among all components of an individual’s life, and could lead to relationships between movement abilities and several other traits. Indeed, and compared to relationships found between movement and life history traits in other groups (in mammals and birds: Bowman 2003, Bowman et al. 2002, Sutherland et al. 2000), we then reported traits that could be related to movement abilities, always in order to help for amphibian management.

## Geographic coverage

### Description

Our database included all amphibian species present in Europe (Frost et al. 2006, IUCN 2011). Four invasive species were included in the database (*Anaxyrus americanus*, *Lithobates catesbeianus*, *Lithobates sylvaticus* and *Xenopus laevis*). A particularity is that *Bufotes viridis* has been recently split in several species. To avoid biased data, we considered all populations to represent identical entities and therefore argued that they shared identical traits. By this way, we reported only traits referring to *Bufotes viridis* (formerly *Bufo viridis*) and we did not take into account traits related to new splited species.

## Taxonomic coverage

### Description

We based our taxonomic coverage on European species described on the IUCN website (IUCN 2011) and from the Amphibian Tree of Life (Frost et al. 2006). More details on species are given in Table 1.

### Taxa included

**Table:** 

Rank	Scientific Name	Common Name
species	Alytes cisternasii	Iberian Midwife Toad
species	Alytes dickhilleni	Betic Midwife Toad
species	Alytes muletensis	Mallorcan Midwife Toad
species	Alytes obstetricans	Common Midwife Toad
species	Anaxyrus americanus	American Toad
species	Atylodes genei	Sardinian Cave Salamander
species	Bombina bombina	Fire-bellied Toad
species	Bombina pachypus	Appenine Yellow-bellied Toad
species	Bombina variegata	Yellow–bellied Toad
species	Bufo bufo	Common Toad
species	Bufo mauritanicus	Mauritanian Toad
species	Calotriton arnoldi	-
species	Calotriton asper	Pyrenean Brook Salamander
species	Chioglossa lusitanica	Golden-striped Salamander
species	Discoglossus galganoi	Iberian Painted Frog
species	Discoglossus jeanneae	Spanish Painted Frog
species	Discoglossus montalentii	Corsican Painted Frog
species	Discoglossus pictus	Painted Frog
species	Discoglossus sardus	Tyrrhenian Painted Frog
species	Epidalea calamita	Natterjack Toad
species	Euproctus montanus	Corsican Brook Salamander
species	Euproctus platycephalus	Sardinian Brook Salamander
species	Hyla arborea	European Tree Frog
species	Hyla intermedia	Italian Tree Frog
species	Hyla meridionalis	Mediterranean Tree Frog
species	Hyla sarda	Tyrrhenian Tree Frog
species	Lissotriton boscai	Iberian Newt
species	Lissotriton helveticus	Palmate Newt
species	Lissotriton italicus	Italian Newt
species	Lissotriton montandoni	Carpathian Newt
species	Lissotriton vulgaris	Smooth Newt
species	Lithobates catesbeianus	American Bullfrog
species	Lithobates sylvaticus	Wood Frog
species	Lyciasalamandra helverseni	-
species	Lyciasalamandra luschani	-
species	Mesotriton alpestris	Alpine Newt
species	Pelobates cultripes	Western Spadefoot
species	Pelobates fuscus	Common Spadefoot
species	Pelobates syriacus	Eastern Spadefoot
species	Pelodytes ibericus	Sapillo Moteado Ibérico
species	Pelodytes punctatus	Parsley Frog
species	Pelophylax bedriagae	Levant Water Frog
species	Pelophylax bergeri	Italian Pool Frog
species	Pelophylax cerigensis	Karpathos Frog
species	Pelophylax cretensis	Cretan Frog
species	Pelophylax epeiroticus	Epirus Water Frog
species	Pelophylax esculentus	Edible Frog
species	Pelophylax grafi	Rana De Graf
species	Pelophylax hispanicus	Italian Edible Frog
species	Pelophylax kurtmuelleri	Balkan Water Frog
species	Pelophylax lessonae	Pool Frog
species	Pelophylax perezi	Perez's Frog
species	Pelophylax ridibundus	Eurasian Marsh Frog
species	Pelophylax shqipericus	Albanian Water Frog
species	Pleurodeles waltl	Sharp-ribbed Salamander
species	Proteus anguinus	Proteus
species	Pseudepidalea viridis	Green Toad
species	Rana arvalis	Altai Brown Frog
species	Rana dalmatina	Agile Frog
species	Rana graeca	Greek Stream Frog
species	Rana iberica	Iberian Frog
species	Rana italica	Italian Stream Frog
species	Rana latastei	Italian Agile Frog
species	Rana pyrenaica	Pyrenean Frog
species	Rana temporaria	European Common Frog
species	Salamandra algira	North African Fire Salamander
species	Salamandra atra	Golden Salamander
species	Salamandra corsica	Corsican Fire Salamander
species	Salamandra lanzai	Lanza's Alpine Salamander
species	Salamandra salamandra	Common Fire Salamander
species	Salamandrina perspicillata	-
species	Salamandrina terdigitata	Spectacled Salamander
species	Speleomantes ambrosii	Ambrosi's Cave Salamander
species	Speleomantes flavus	Monte Albo Cave Salamander
species	Speleomantes imperialis	Imperial Cave Salamander
species	Speleomantes italicus	Italian Cave Salamander
species	Speleomantes sarrabusensis	-
species	Speleomantes strinatii	North-west Italian Cave Salamander
species	Speleomantes supramontis	Supramonte Cave Salamander
species	Triturus carnifex	Italian Crested New
species	Triturus cristatus	Northern Crested Newt
species	Triturus dobrogicus	Danube Crested Newt
species	Triturus karelinii	Southern Crested Newt
species	Triturus marmoratus	Marbled Newt
species	Triturus pygmaeus	Southern Marbled Newt
species	Xenopus laevis	Platanna

## Traits coverage

### *Morphological traits*

We reported 14 morphological traits (32 modalities) for each European amphibian species: body lengths, body mass, limb lengths and details about webbing and fingers (Table 3). These traits were supposed to be relevant for amphibian conservation.

### *Life history traits*

We collected 17 life history traits (65 modalities), when available, for European amphibians (Table 4). Life history traits vary considerably between species, and between Anura and Urodela in particular. The database includes data about activity, survival rates, sexual maturity, mating systems, characteristics of eggs and clutch position, parental care, foot diet, defense system, communication and territoriality.

### *Movement*

**Figure 2.**
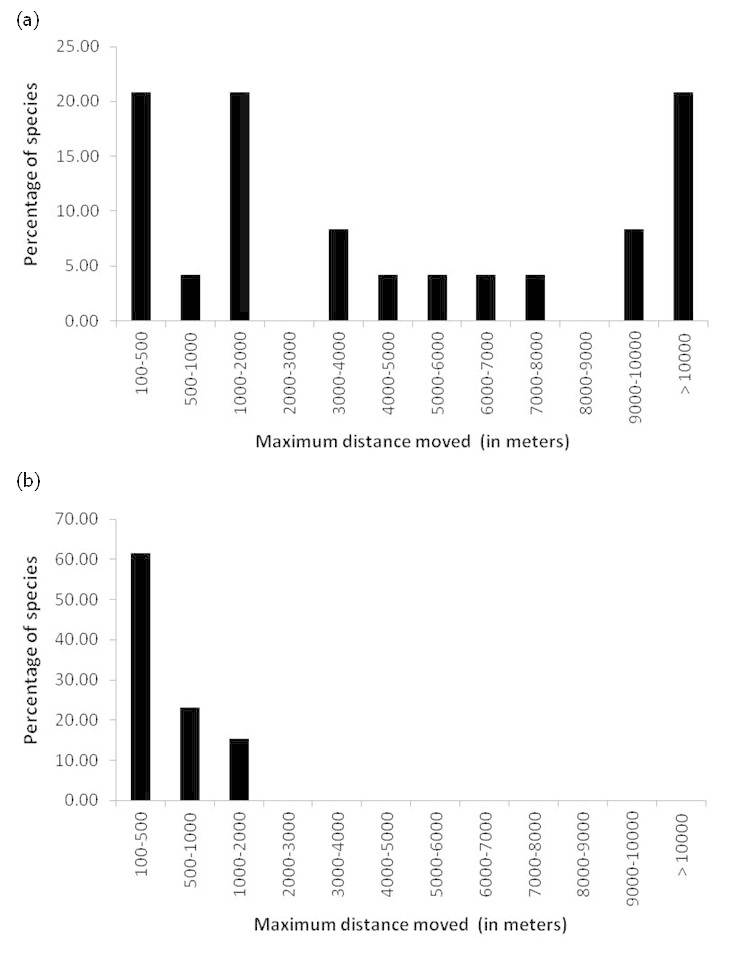
Frequency histogram of the maximum distance moved by (a) 56 Anura and (b) 30 Urodela species.

We reported 7 traits related to movement, by separating when possible migration (N subcategories) and dispersal events (21 modalities; Table 5). In contrast to migration, dispersal is defined as individual movement that induces gene flow (Ronce 2007). Amphibians regularly migrate between terrestrial and aquatic habitats, and some individuals also engage in dispersal, leaving their population of birth (or previous reproduction) to join another suitable habitat in the landscape. We used the maximum distance (and not the modal distance) recorded by species because long-distance dispersal movements have considerably higher impact on species spread, species persistence, and metapopulation functioning (Trakhtenbrot et al. 2005). All dispersal and migration values were estimated using mark-release-recapture or individual tracking. In general, amphibians are considered as low dispersal species (Boissinot 2009, Smith and Green 2005), but we detected several species for which dispersal and/or migration distances were > 10 km (Fig. 2).

### *Habitat preferences and distribution*

We collected habitat preferences and 2 traits related to spatial distribution (113 modalities; Table 6). Amphibians are often considered as specialized to certain habitat types, which make them particularly sensitive to landscape changes. Nevertheless, habitat preferences, and particularly terrestrial habitats of amphibian species request much clarification, especially given the recent changes induced by habitat fragmentation. We categorized habitats as follows (IUCN 2011): forest, savanna, shrubland, grassland, wetlands, rocky areas, caves and subterranean habitats (non-aquatic), deserts, artificial/terrestrial habitats, and other. We chose to use the following IUCN habitats because in our opinion it is the most conservative assumptions about species delimitations and also because these are the entities currently recognized by international conservation authorities and that is the actual aim of the database. Moreover, for this inter-specific database we avoided selecting too specific habitats, and chose relatively broad habitat categories which included all habitats in which species live. We also noted biogeographical regions (European Environment Agency 2010) where species were present, and the proportion of their distribution map on each continent.

### *Threats*

We collected information on three categories (22 modalities; Table 7) related to threats of amphibian species: The IUCN status (6 subcategories), population trend (4 subcategories), and major threats (12 subcategories).

## Usage rights

### Use license

Creative Commons CCZero

## Data resources

### Data package title

European amphibians database

### Number of data sets

1

### Data set 1.

#### Data set name

Database fo life-history traits for European amphibians

#### Data format

xls

#### Number of columns

45

#### Description

Summary of morphometric and life-history traits for 86 European amphibian species (Suppl. material 1). Values expected for the life-histories have been averaged between studies (i.e. between populations). DD (data deficient) means that data were not available in the literature.

**Data set 1.** 

Column label	Column description
Sexual dimorphism	Sexual dimorphism
Body mass (in g)	Body mass (in g)
Snout-to-vent length (in mm)	Snout-to-vent length (in mm)
Total length (in mm)	Total length (in mm)
Proportion head length/body length	Proportion head length/body length
Foot length (in mm)	Foot length (in mm)
Hind limb length (in mm)	Hind limb length (in mm)
Tibia length (in mm)	Tibia length (in mm)
Proportion forelimb/hindlimb length	Proportion forelimb/hindlimb length
Discs	Discs
Webbing	Webbing
Number of toes/fingers	Number of toes/fingers
Tubercle	Tubercle
Coloration	Coloration
Activity	Activity
Survival rates	Survival rates
Sexual maturity (in years)	Sexual maturity (in years)
Mating systems	Mating systems
Number of eggs/offspring	Number of eggs/offspring
Egg laying mode	Egg laying mode
Eggs and larvae characteristics	Eggs and larvae characteristics
Clutch position	Clutch position
Breeding season	Breeding season
Parental care	Parental care
Active or passive feeding	Active or passive feeding
Food of juveniles	Food of juveniles
Food of adults	Food of adults
Metabolism	Metabolism
Defense	Defense
Communication	Communication
Territoriality	Territoriality
Home range	Home range
Movement event	Movement event
Dispersal active or passive	Dispersal active or passive
Sex biased dispersal	Sex biased dispersal
Mode of displacement	Mode of displacement
Dispersal	Dispersal
Migration	Migration
Habitat	Habitat
Topography	Topography
Biogeographical region	Biogeographical region
Distribution	Distribution
IUCN status	IUCN status
Population trend	Population trend
Major threats	Major threats

## Additional information

**Conclusion**

Our database is the first comprehensive trait database in European amphibians. After an extensive research effort, our database highlighted the lack of data about amphibian traits and more generally, on amphibian’s biology. Improve our knowledge on amphibians should certainly help for their management, which might strongly enhance their conservation plans. Morphological traits, which are easy to collect, are still unavailable for many species. Data about movement abilities (both dispersal and migration) were the least informed data of all database. In particular, we showed that movement traits, which are difficult to collect, were unknown for a majority of threatened species. This database could be an essential support for management and conservation plans, and should be more efficient when all data will be available.

## Supplementary Material

Supplementary material 1 Database for life history traits for European amphibians Data type: life history traits
Brief description: Summary of morphometric and life-history traits for 86 European amphibian species. Values expected for the life-histories have been averaged between studies (i.e. between populations). NA means that data were not available in the literature.
File: oo_32985.xlsx Trochet A, Moulherat S, Calvez O, Schmeller DS, Clobert J, Stevens VM

Supplementary material 2 References cited in the database for life-history traits for European amphibians Data type: xls
File: oo_32986.xlsx Trochet A, Moulherat S, Calvez O, Schmeller DS, Clobert J, Stevens VM

## Figures and Tables

**Table 1.** Details on species used in our database. For each species, synonyms and family were given. Information about spatial distribution (if the species is an invasive species or not, and if it lives out of Europe) was reported. List of references associated (Suppl. material 2), number of publications used and the percent of missing values for each species were also given.

Anurans/ Urodela	Species	Synonyms	Family	Invasive Species (Yes/No)	Species living out of Europe	References	Number of publications used	% of missing values
A	*Alytes cisternasii*	-	Discoglossidae	No	No	1, 2, 3, 4, 5, 6, 7	7	11.46
A	*Alytes dickhilleni*	-	Discoglossidae	No	No	4, 8	2	26.09
A	*Alytes muletensis*	-	Discoglossidae	No	No	4, 7, 9	3	21.34
A	*Alytes obstetricans*	-	Discoglossidae	No	No	2, 3, 5, 6, 10, 11, 12, 13, 14, 15, 16, 17	12	5.14
A	*Anaxyrus americanus*	*Bufo americanus*	Bufonidae	Yes	Yes	7, 137, 228, 229	4	13.83
U	*Atylodes genei*	-	Plethodontidae	No	No	4, 138, 139, 140, 141, 142	6	22.53
A	*Bombina bombina*	-	Bombinatoridae	No	Yes	4, 6, 18, 19, 20, 21	6	3.56
A	*Bombina pachypus*	-	Bombinatoridae	No	No	6, 19	2	50.59
A	*Bombina variegata*	-	Bombinatoridae	No	No	4, 11, 13, 21, 22, 23, 24, 25, 26, 27, 28, 29, 30, 31, 32, 33	16	7.11
A	*Bufo bufo*	-	Bufonidae	No	Yes	4, 5, 6, 11, 13, 14, 15, 19, 26, 30, 34, 35, 36, 37, 38, 39, 40, 41, 42, 43, 44, 48, 49, 50	24	1.98
A	*Bufo mauritanicus*	-	Bufonidae	No	Yes	12, 48	2	30.83
U	*Calotriton arnoldi*	-	Salamandridae	No	No	139, 142, 143	3	54.15
U	*Calotriton asper*	*Euproctus asper*	Salamandridae	No	No	4, 11, 13, 139, 140, 141, 144, 145, 146, 147, 148, 149, 150, 151	14	7.91
U	*Chioglossa lusitanica*	-	Salamandridae	No	No	4, 139, 140, 141, 152, 156, 157, 158	8	10.28
A	*Discoglossus galganoi*	*Discoglossus hispanicus*	Discoglossidae	No	No	4, 7, 49	3	15.81
A	*Discoglossus jeanneae*	-	Discoglossidae	No	No	4, 50, 51	3	24.11
A	*Discoglossus montalentii*	-	Discoglossidae	No	No	11, 52	2	22.92
A	*Discoglossus pictus*	-	Discoglossidae	No	Yes	4, 6, 11, 53, 54, 55	6	13.44
A	*Discoglossus sardus*	-	Discoglossidae	No	No	4, 11, 52, 56, 57	5	18.97
A	*Epidalea calamita*	*Bufo calamita*	Bufonidae	No	Yes	4, 11, 13, 15, 25, 26, 56, 58, 59, 60, 61, 62, 63, 66	14	1.19
U	*Euproctus montanus*	-	Salamandridae	No	No	4, 7, 139, 141, 157	5	17.79
U	*Euproctus platycephalus*	-	Salamandridae	No	No	4, 139, 141, 157, 158	5	13.44
A	*Hyla arborea*	-	Hylidae	No	Yes	4, 5, 11, 13, 15, 58, 66, 67, 68, 69, 70, 71, 72, 73	14	2.37
A	*Hyla intermedia*	*Hyla italica*	Hylidae	No	No	4, 57, 58, 67, 74	5	39.13
A	*Hyla meridionalis*	-	Hylidae	No	Yes	7, 11, 12, 13, 58, 75	6	9.88
A	*Hyla sarda*	-	Hylidae	No	No	11, 26, 57, 67	4	13.04
U	*Lissotriton boscai*	*Triturus boscai*	Salamandridae	No	No	4, 139, 140, 141, 159, 161, 162, 163, 164	9	15.02
U	*Lissotriton helveticus*	*Triturus helveticus*	Salamandridae	No	No	4, 11, 13, 15, 26, 59, 139, 140, 141, 150, 163, 164, 165, 166	14	7.91
U	*Lissotriton italicus*	*Triturus italicus*	Salamandridae	No	No	4, 139, 159, 167	4	18.18
U	*Lissotriton montandoni*	*Triturus montandoni*	Salamandridae	No	No	4, 139, 140, 141, 159, 168, 169	7	18.18
U	*Lissotriton vulgaris*	*Triturus vulgaris*	Salamandridae	No	Yes	4, 11, 14, 15, 18, 19, 26, 44, 59, 139, 140, 141, 150, 164, 169, 172, 173, 174, 175, 176, 177, 178, 179, 180, 181, 182, 183, 184	28	5.53
A	*Lithobates catesbeianus*	*Rana catesbeiana*	Ranidae	Yes	Yes	4, 5, 11, 13, 15, 26, 76, 77, 78, 79, 80, 81	12	4.35
A	*Lithobates sylvaticus*	*Rana sylvatica*	Ranidae	Yes	Yes	5, 7, 230, 231, 232	5	13.44
U	*Lyciasalamandra helverseni*	*Mertensiella luschani helverseni*	Salamandridae	No	No	139, 182	2	49.80
U	*Lyciasalamandra luschani*	*Mertensiella luschani*	Salamandridae	No	Yes	4, 140, 184, 185, 186, 187	6	14.62
U	*Mesotriton alpestris*	*Triturus alpestris*, *Ichthyosaura alpestris*	Salamandridae	No	No	4, 11, 14, 15, 19, 26, 59, 140, 141, 142, 151, 164, 165, 176, 188, 189, 190, 191, 192	19	6.72
A	*Pelobates cultripes*	-	Pelobatidae	No	No	4, 6, 11, 13, 56, 82, 83	7	11.46
A	*Pelobates fuscus*	-	Pelobatidae	No	Yes	4, 5, 6, 11, 19, 26, 84, 85, 86, 87	10	5.14
A	*Pelobates syriacus*	*Pelobates transcaucasicus*	Pelobatidae	No	Yes	4	1	17.00
A	*Pelodytes ibericus*	-	Pelodytidae	No	No	4, 88, 89	3	17.79
A	*Pelodytes punctatus*	-	Pelodytidae	No	No	4, 11, 13, 15, 58, 89	6	13.04
A	*Pelophylax bedriagae*	*Rana bedriagae*	Ranidae	No	Yes	4, 90	2	23.32
A	*Pelophylax bergeri*	*Rana bergeri*	Ranidae	No	No	4	1	22.13
A	*Pelophylax cerigensis*	*Rana cerigensis*	Ranidae	No	No	4, 91	2	31.23
A	*Pelophylax cretensis*	*Rana cretensis*	Ranidae	No	No	4, 92	2	31.23
A	*Pelophylax epeiroticus*	*Rana epeirotica*	Ranidae	No	No	4, 93, 94, 95, 96	5	24.51
A	*Pelophylax esculentus*	*Rana esculenta*	Ranidae	No	Yes	4, 6, 14, 15, 18, 97, 98, 99, 100, 101, 102	11	5.14
A	*Pelophylax grafi*	*Rana grafi*	Ranidae	No	No	4, 26, 97	3	23.72
A	*Pelophylax hispanicus*	*Rana hispanica*	Ranidae	No	No	4, 51	2	51.78
A	*Pelophylax kurtmuelleri*	*Rana kurtmuelleri*	Ranidae	No	No	4, 51	2	28.85
A	*Pelophylax lessonae*	*Rana lessonae*	Ranidae	No	Yes	4, 6, 14, 15, 26, 97, 98, 99, 100, 101, 103, 104, 105	13	4.35
A	*Pelophylax perezi*	*Rana perezi*	Ranidae	No	No	4, 5, 26, 97, 106	5	15.81
A	*Pelophylax ridibundus*	*Rana ridibunda*	Ranidae	No	Yes	4, 14, 15, 58, 97, 99, 100, 102, 107, 108, 109, 110, 111, 112	14	3.95
A	*Pelophylax shqipericus*	*Rana shqiperica*	Ranidae	No	No	4, 113	2	34.78
U	*Pleurodeles waltl*		Salamandridae	No	Yes	4, 7, 12, 140, 141, 142, 177, 193	8	13.83
U	*Proteus anguinus*	*Siren anguina*	Proteidae	No	No	4, 140, 141, 142, 148, 149, 194	7	14.23
A	*Pseudepidalea viridis*	*Bufo viridis*, *Bufotes viridis*	Bufonidae	No	Yes	4, 11, 12, 26, 30, 51, 58, 90, 115, 116, 117, 118, 119	13	5.93
A	*Rana arvalis*	-	Ranidae	No	Yes	4, 11, 15, 19, 58, 120	6	6.72
A	*Rana dalmatina*	*Rana agilis*	Ranidae	No	Yes	4, 11, 13, 15, 30, 46, 58, 121, 122, 123	10	3.56
A	*Rana graeca*	-	Ranidae	No	No	4, 58	2	12.65
A	*Rana iberica*	-	Ranidae	No	No	4, 5, 58, 123	4	12.25
A	*Rana italica*	-	Ranidae	No	No	4, 51	2	23.72
A	*Rana latastei*	-	Ranidae	No	No	4, 58, 124, 125	4	15.02
A	*Rana pyrenaica*	-	Ranidae	No	No	4, 11, 13, 26, 126, 127	6	17.39
A	*Rana temporaria*	-	Ranidae	No	Yes	4, 5, 11, 13, 14, 15, 19, 26, 44, 58, 120, 121, 126, 128, 129, 130, 131, 132, 133	19	2.37
U	*Salamandra algira*	-	Salamandridae	No	Yes	12, 140	2	32.02
U	*Salamandra atra*	-	Salamandridae	No	No	4, 11, 140, 141, 142, 151, 195, 196	8	18.58
U	*Salamandra corsica*	-	Salamandridae	No	No	26, 140, 197	3	22.92
U	*Salamandra lanzai*	-	Salamandridae	No	No	11, 26, 140, 198, 199, 200	6	17.79
U	*Salamandra salamandra*	-	Salamandridae	No	No	11, 13, 14, 15, 19, 140, 141, 142, 151, 200, 201, 202, 203, 204, 205	15	9.88
U	*Salamandrina perspicillata*	-	Salamandridae	No	No	140, 142, 206, 207, 208, 209	6	58.89
U	*Salamandrina terdigitata*	*Molge tridactyla*	Salamandridae	No	No	4, 140, 141, 208	4	24.51
U	*Speleomantes ambrosii*	-	Plethodontidae	No	No	4, 139, 140	3	14.62
U	*Speleomantes flavus*	-	Plethodontidae	No	No	4, 139, 140	3	39.13
U	*Speleomantes imperialis*	-	Plethodontidae	No	No	4, 139, 140	3	17.00
U	*Speleomantes italicus*	-	Plethodontidae	No	No	4, 139, 140, 141	4	16.21
U	*Speleomantes sarrabusensis*	-	Plethodontidae	No	No	4	1	44.27
U	*Speleomantes strinatii*	-	Plethodontidae	No	No	4, 11, 26, 140, 211	5	15.02
U	*Speleomantes supramontis*	-	Plethodontidae	No	No	4, 139, 140	3	39.13
U	*Triturus carnifex*	-	Salamandridae	No	No	4, 18, 36, 140, 160, 176, 197, 212, 213, 214	10	8.70
U	*Triturus cristatus*	-	Salamandridae	No	Yes	4, 11, 15, 18, 19, 26, 44, 56, 59, 140, 141, 142, 151, 160, 165, 173, 174, 175, 176, 177, 181, 215, 216, 217, 218, 219	26	4.35
U	*Triturus dobrogicus*	-	Salamandridae	No	No	4, 18, 85, 140, 160, 176, 177, 220	8	26.09
U	*Triturus karelinii*	-	Salamandridae	No	Yes	4, 140, 142, 160, 176, 177, 221, 222, 223	9	40.71
U	*Triturus marmoratus*	-	Salamandridae	No	No	4, 11, 13, 18, 26, 56, 140, 142, 160, 164, 215, 216, 217, 224, 225	15	10.67
U	*Triturus pygmaeus*	-	Salamandridae	No	No	4, 140, 141, 160, 224, 226, 227	7	18.18
A	*Xenopus laevis*	*Bufo laevis*	Pipidae	Yes	Yes	5, 11, 26, 134, 135, 136	6	11.86

**Table 2.** Mean and range (min-max) of several traits recorded in Anura and Urodela species from our database. Number of missing values is also reported.

	Anura (N=50)			Urodela (N=36)		
	Mean	Range (min–max)	Number of missing values	Mean	Range (min–max)	Number of missing values
Body mass (in g)	32.34	2.31–307.23	19	6.68	0.98–35.23	10
Snout-to-vent length in adults (in mm)	61.89	35.18–141.00	0	63.64	33.31–169.90	4
Snout-to-vent length in males (in mm)	56.01	34.70–134.74	9	59.19	31–129.75	7
Snout-to-vent length in females (in mm)	62.14	35.65–150.00	10	61.72	38.12–155.25	7
Total length in adults (in mm)	61.89	35.18–141.00	0	126.15	67.28–257.00	1
Foot length (in mm)	27.73	5.37–65.95	25	7.56	6.50–9.09	31
Tibia length (in mm)	26.89	13.15–56.81	19	18.88	4.81–32.94	34
Hind limb length (in mm)	90.40	45.28–188.98	25	19.71	10.68–41.00	8
Metamorphosis size (in mm)	20.83	9.50–95.00	14	38.76	20.00–70.00	5
Number of eggs	4875.70	20–25000	0	164.06	2–1400	0
Survival rates in adults	0.64	0.34–0.80	44	0.63	0.42–0.79	27
Sexual maturity (in years)	2.18	1–4	12	3.35	1.5–7	8
Movement ability (in m)	5422.73	150–15000	26	481.08	21–1500	23

**Table 3.** Definition of the 14 morphological traits and their modalities in the European amphibian database. For all traits recorded, DD (data deficient) means that no data were reported. When several values were available for a trait, we averaged them across studies (i.e. between populations).

**Sexual dimorphism**	Difference in ornamentation (coloration) or in size (length of tail, size of head or body size) between sexes. In amphibians, females are generally bigger than males. This difference may be caused by natural selection of a large female size due to a fecundity advantage. This phenotypic difference in size is often explained by sexual selection.
Modalities:	
0	Absence of sexual dimorphism.
1	Presence of sexual dimorphism.
**Body mass**	Body mass in males, females or both when sex specific data were not available.
Modalities:	
Body mass in males	Body mass in males, in grams.
Body mass in females	Body mass in males, in grams.
Adult body mass	Body mass in adults, without distinction between males and females, in grams. This data was recorded when no sex specific data on body mass were available in the literature.
Body mass in juveniles	Body mass in juveniles, in grams. No distinction between sexes was available.
***Body length***	A measurement of the longest dimension of a body, typically between two distinct ends of the body. In amphibians, the distance snout-to-vent length is usually measured. To take into account the tail length in Urodela, we also reported a total length for each species, in males, in females or both when sex-specific data were not available.
**Snout-to-vent length**	
Modalities:	
Snout-to-vent length in males	Measurement between the snout and the vent in males (in millimeters).
Snout-to-vent length in females	Measurement between the snout and the vent in females (in millimeters).
Adult snout-to-vent length	Measurement between the snout and the vent in adults, when no sex specific data were available (in millimeters).
**Total length**	In Anura, this measure was similar to the snout-to-vent length. In Urodela, this measurement takes into account the tail length.
Modalities:	
Total length in males	Measure of the total body length in males, in millimeters.
Total length in females	Measure of the total body length in females, in millimeters.
Adult total length	Measure of the total body length in adults, when no sex specific data were available, in millimeters.
**Head and body length proportion**	Proportion of the head length compared to the body length.
Modalities:	
Head length < Body length	0: Head length is not lower than body length. 1: Head length is lower than body length.
Head length = Body length	0: Head length is not similar to body length. 1: Head length is similar to body length.
Head length > Body length	0: Head length is not longer than body length. 1: Head length is longer than body length.
***Limb length***	Measurements of limb (foot, tibia and hind limb) in males, in females, or both when data were not sex specific available.
**Foot length**	Measurement of the foot in millimeters.
Modalities:	
Foot length in males	Measurement of the foot in males, in millimeters.
Foot length in females	Measurement of the foot in females, in millimeters.
Adult foot length	Measurement of the foot in adults, when no sex specific data were available, in millimeters.
**Hind limb length**	Measurement of the hind limb, in millimeters.
Modalities:	
Hind limb length in males	Measurement of the hind limb in males, in millimeters.
Hind limb length in females	Measurement of the hind limb in females, in millimeters.
Adult hind limb length	Measurement of the hind limb in adults, when no sex specific data were available, in millimeters.
**Tibia length**	Measurement of the tibia, in millimeters.
Modalities:	
Tibia length in males	Measurement of the tibia in males, in millimeters.
Tibia length in females	Measurement of the tibia in females, in millimeters.
Adult tibia length	Measurement of the tibia in adults, when no sex specific data were available, in millimeters.
**Fore and hind limb proportion**	Proportion of the fore limb length compared to the hind limb length.
Modalities:	
Fore limb length < Hind limb length	0: Fore limb length is not lower than hind limb length. 1: Fore limb length is lower than hind limb length.
Fore limb length = Hind limb length	0: Fore limb length is not similar to hind limb length. 1: Fore limb length is similar to hind limb length.
Fore limb length > Hind limb length	0: Fore limb length is not longer than hind limb length. 1: Fore limb length is longer than hind limb length.
***Fingers and webbing***	
**Presence of discs**	Some amphibians have adhesive discs at the ends of the toes and fingers.
Modalities:	
0	Absence of adhesive discs
1	Presence of adhesive discs on fingers and/or on toes.
**Webbing**	
Modalities:	
Presence of webbing	0: Absence of webbing. 1: Presence of webbing on toes and/or fingers
Presence of webbing on toes only	0: Absence of webbing on toes only. 1: Presence of webbing on toes only.
Presence of webbing on toes and fingers	0: Absence of webbing on toes and fingers. 1: Presence of webbing on toes and fingers.
**Number of toes/fingers**	Most of amphibians have 5 toes on their feet and 4 fingers on their hands. But some of them can have less than 5 toes and/or 4 fingers. We reported if each species have a reduction only on fingers (4), or if the individuals have less than 5 toes and 4 fingers.
Modalities:	
Reduction on fingers only	0: Individuals have less than 5 toes and 4 fingers. 1: Individuals have 5 toes and 4 fingers.
Reduction on fingers and toes	0: Individuals have 5 toes and 4 fingers. 1: Individuals have less than 5 toes and 4 fingers.
**Presence of metatarsal tubercle**	Some species have a metatarsal tubercle on hind limb. A prominent inner metatarsal tubercle used for burrowing with the hind limbs. When available, the length of the tubercle is given.
Modalities:	
0	Absence of metatarsal tubercle.
1	Presence of metatarsal tubercle.
**Coloration**	
Modalities:	
Dorsoseparation	0: Dorsoseparation weak 1: Dorsoseparation sharp
Webbing colour contrast	0: Absence of webbing colour contrast 1: Presence of webbing colour contrast

**Table 4.** Definition of the 17 life history traits and their modalities in the European amphibian database. For all traits recorded, DD (data deficient) means that none data were reported in the literature. When several values were available for a trait, we averaged them across studies (i.e. between populations).

**Activity**	Details about the period of activity.
Modalities:	
Diurnal	0: No diurnal species. 1: Diurnal species.
Nocturnal	0: No nocturnal species. 1: Nocturnal species.
Both	0: Species diurnal or nocturnal. 1: Species diurnal and nocturnal.
**Survival rates**	Survival rate indicates the percentage of individuals who are alive for a given period of time.
Modalities:	
Survival rates in males	Survival rates in males.
Survival rates in females	Survival rates in females.
Adult survival rates	Survival rates in adults, when no sex specific data were available.
**Sexual maturity**	Sexual maturity in years.
Modalities:	
Sexual maturity in males	Sexual maturity in males, in years.
Sexual maturity in females	Sexual maturity in females, in years.
Adult sexual maturity	Sexual maturity in adults, when no sex specific data were available, in years.
**Mating systems**	Structuration of sexual behaviour relationships during the breeding season. We recorded 2 different types of mating systems through amphibians: polygyny when a male has mating relationships with several females; polyandry when a female has mating relationships with several males.
Modalities:	
Polyandry	0: No polyandry species. 1: Polyandry species
Polygyny	0: No polygyny species. 1: Polygyny species.
***Eggs and young***	Details about clutch size, egg laying mode, eggs and young characteristics.
**Number of eggs/offspring**	
Modalities:	
Viviparous: number of offspring	When the species is viviparous, number of offspring by female by clutch.
Ovoviviparous: number of eggs	When the species is ovoviviparous, number of eggs by female by clutch.
Ovoviviparous: number of offspring	When the species is ovoviviparous, number of offspring by female by clutch.
**Egg laying mode**	
Modalities:	
Single	0: Eggs are not laid single. 1: Eggs are laid single.
Cluster	0: Eggs are not laid by cluster. 1: Eggs are laid by cluster.
Strings	0: Eggs are not laid by strings. 1: Eggs are laid by strings.
**Eggs and larvae characteristics**	
Modalities:	
Metamorphosis size	Measurement of the total body length of juveniles before metamorphosis, in millimeters.
Number of eggs	When the species is oviparous, number of eggs by female by clutch.
Egg diameter	Egg diameter in millimeters.
Egg mass	Egg mass in grams.
Pole visible on eggs	0: Pole not visible on eggs. 1: Pole visible on eggs.
**Clutch position**	Eggs can be lay at different places in the breeding environment.
Modalities:	
On ground	0: Eggs are not laid on ground. 1: Eggs are laid on ground.
On adult	0: Eggs are not laid on adult. 1: Eggs are laid on adult.
Attach in lotic habitat	0: Eggs are not attached in lotic habitat. 1: Eggs are attached in lotic habitat.
Attach in lentic habitat	0: Eggs are not attached in lentic habitat. 1: Eggs are attached in lentic habitat.
Free in lentic habitat	0: Eggs are not laid free in lentic habitat. 1: Eggs are laid free in lentic habitat.
On surface in lentic habitat	0: Eggs are not laid on surface in lentic habitat. 1: Eggs are laid on surface in lentic habitat.
Attach on swamps in lentic habitat	0: Eggs are not attached on swamps in lentic habitat. 1: Eggs are attached on swamps in lentic habitat.
Free on swamps in lentic habitat	0: Eggs are not laid free on swamps in lentic habitat. 1: Eggs are laid free on swamps in lentic habitat.
On surface on swamps in lentic habitat	0: Eggs are not laid on surface on swamps in lentic habitat. 1: Eggs are laid on surface on swamps in lentic habitat.
On surface on non-permanent lentic ponds	0: Eggs are not laid on surface on non-permanent lentic ponds. 1: Eggs are laid on surface on non-permanent lentic ponds.
**Breeding season**	Breeding season can be prolonged or explosive (*breeding* periods of a few days to a few *weeks).*
Modalities:	
Explosive or prolonged	0: Prolonged breeding season. 1: Explosive breeding season.
**Parental care**	Parental care is defined as any behaviour of parents for increasing the fitness of their young. Most of amphibians do not perform parental care, but a few transport, guard and defend their eggs.
Modalities:	
Presence of parental care	0: Absence of parental care. 1: Presence of parental care.
Transport of eggs	0: Parents do not transport their eggs. 1: Parents transport their eggs.
Transport of tadpoles	0: Parents do not transport their tadpoles. 1: Parents transport their tadpoles.
Guarding eggs	0: Parents do not guard their eggs. 1: Parents guard their eggs.
Defend eggs	0: Parents do not defend their eggs. 1: Parents defend their eggs.
Water eggs	0: Parents do not humidify their eggs. 1: Parents humidify their eggs.
***Food diet***	
**Food active or passive**	
Modalities:	
0	Food passive (sit-and-wait)
1	Food active (active foragers)
**Food of juveniles**	
Modalities:	
Carnivorous	0: Juveniles are not carnivorous. 1: Juveniles are carnivorous.
Insectivorous	0: Juveniles are not insectivorous. 1: Juveniles are insectivorous.
Moluscivorous	0: Juveniles are not moluscivorous. 1: Juveniles are moluscivorous.
Cannibalism	0: Juveniles are not cannibals. 1: Juveniles are cannibals.
Herbivorous	0: Juveniles are not herbivorous. 1: Juveniles are herbivorous.
Detritivorous	0: Juveniles are not detritivorous. 1: Juveniles are detritivorous.
**Food of adults**	
Modalities:	
Carnivorous	0: Adults are not carnivorous. 1: Juveniles are carnivorous.
Insectivorous	0: Juveniles are not insectivorous. 1: Juveniles are insectivorous.
Moluscivorous	0: Juveniles are not moluscivorous. 1: Juveniles are moluscivorous.
Cannibalism	0: Juveniles are not cannibals. 1: Juveniles are cannibals.
Herbivorous	0: Juveniles are not herbivorous. 1: Juveniles are herbivorous.
Detritivorous	0: Juveniles are not detritivorous. 1: Juveniles are detritivorous.
**Metabolism**	
Modalities:	
Metabolism rates	Metabolism rate in adults.
**Defense**	Mode of defense
Modalities:	
Secretion	0: Species do not use secretion as mode of defense. 1: Species uses secretion as mode of defense.
Toxicity	0: Species do not use toxicity as mode of defense. 1: Species uses toxicity as mode of defense.
Death simulation	0: Species do not use death simulation as mode of defense. 1: Species uses death simulation as mode of defense.
Unken reflex	0: Species do not use unken reflex as mode of defense. 1: Species uses unken reflex as mode of defense.
Other	0: Species do not use other mode of defense. 1: Species uses other mode of defense.
**Communication**	Mode of communication
Modalities:	
Visual	0: Species do not use visual communication. 1: Species uses visual communication.
Acoustic	0: Species do not use acoustic communication. 1: Species uses acoustic communication.
Chemical	0: Species do not use chemical communication. 1: Species uses chemical communication.
Seismic	0: Species do not use seismic communication. 1: Species uses seismic communication.
**Territoriality**	Territoriality can serve individuals to defend their nest, den, sexual partners, mating sites or high quality resources sites needed for themselves or their young. We recorded species for which individuals develop a territorial behaviour (by scent markings or fighting) during the breeding season.
Modalities:	
Male territorial	0: No. 1: Yes.
Female territorial	0: No. 1: Yes.
Both territorial	0: Both males and females not territorial. 1: Both males and females territorial.

**Table 5.** Definition of the 7 traits related to movement in the European amphibian database. For all traits recorded, DD (data deficient) means that no data were reported in the literature. When several values were available for a trait, we averaged them across studies (i.e. between populations).

**Home range**	
Modalities:	
Home range	Here, we consider home range as the area that an individual needs throughout a year. Home range recorded in the literature, in m².
**Movement event**	
Modalities:	
Movement event	0: Movement events performed by one single individual (solitary individual). 1: Movement events performed by several individuals (social individuals).
**Dispersal active or passive**	
Modalities:	
Dispersal active or passive	0: Passive dispersal. 1: Active dispersal.
**Sex-biased dispersal**	Dispersal abilities can be significantly different between genders. We reported here if sex-biased dispersal was identified in species (if males have faster or longer dispersal abilities than females).
Modalities:	
Sex-biased dispersal	0: No sex-biased dispersal reported in the literature. 1: Significant sex-biased dispersal reported in the literature.
**Mode of displacement**	
Modalities:	
Walker	0: Individuals do not walk during displacement event. 1: Individuals walk during displacement event.
Jumper	0: Individuals do not jump during displacement event. 1: Individuals jump during displacement event.
Runner	0: Individuals do not run during displacement event. 1: Individuals run during displacement event.
Climber	0: Individuals do not climb during displacement event. 1: Individuals climb during displacement event.
Swimmer	0: Individuals do not swim during displacement event. 1: Individuals swim during displacement event.
Crawler	0: Individuals do not crawl during displacement event. 1: Individuals crawl during displacement event.
**Dispersal**	Contrary to migration, dispersal is defined as individual movement could induce gene flow. Dispersal distances came from mark-release-recapture and/or radio-tracking studies.
Modalities:	
Dispersal stage juvenile	0: Dispersal event not at juvenile stage. 1: Dispersal event at juvenile stage.
Dispersal stage adult	0: Dispersal event not at adult stage. 1: Dispersal event at adult stage.
Dispersal stage during breeding season	0: Dispersal event not during breeding season. 1: Dispersal event during breeding season.
Mean dispersal distance	Mean dispersal distance, in meters.
Maximum dispersal distance	Maximum dispersal distance, in meters.
Minimal dispersal distance	Minimal dispersal distance, in meters.
**Migration**	Contrary to dispersal, migration is defined as individual movement that not induces gene flow. Migration distances came from mark-release-recapture and/or radio-tracking studies.
Modalities:	
Migration stage adult	0: Migration event not at adult stage. 1: Migration event at adult stage.
Migration stage during breeding season	0: Migration event not during breeding season. 1: Migration event during breeding season.
Mean migration distance	Mean migration distance, in meters.
Maximum migration distance	Maximum migration distance, in meters.
Minimal migration distance	Minimal migration distance, in meters.

**Table 6.** Definition of the habitat preferences and the 2 traits related to the spatial distribution in the European amphibian database (IUCN 2011). For all traits recorded, DD (data deficient) means that no data were reported in the literature.

**Habitat**	Habitat(s) where the species is frequently found.
Modalities:	
*Forest*	
Boreal	0: The species does not live in boreal forests. 1: The species lives in boreal forests.
Subarctic	0: The species does not live in subarctic forests. 1: The species lives in subarctic forests.
Subantarctic	0: The species does not live in subantarctic forests. 1: The species lives in subantarctic forests.
Subtropical/tropical dry	0: The species does not live in subtropical/tropical dry forests. 1: The species lives in subtropical/tropical dry forests.
Temperate	0: The species does not live in temperate forests. 1: The species lives in temperate forests.
Subtropical/tropical moist lowland	0: The species does not live in subtropical/tropical moist lowland forests. 1: The species lives in subtropical/tropical moist lowland forests.
Subtropical/tropical mangrove vegetation aboral	0: The species does not live in subtropical/tropical mangrove vegetation aboral forests. 1: The species lives in subtropical/tropical mangrove vegetation aboral forests.
Subtropical/tropical swamp	0: The species does not live in subtropical/tropical swamp forests. 1: The species lives in subtropical/tropical swamp forests.
Subtropical/tropical moist montane	0: The species does not live in subtropical/tropical moist montane forests. 1: The species lives in subtropical/tropical moist montane forests.
*Savanna*	
Dry	0: The species does not live in dry savanna. 1: The species lives in dry savanna.
Moist	0: The species does not live in moist savanna. 1: The species lives in moist savanna.
*Shrubland*	
Subarctic	0: The species does not live in subarctic shrubland. 1: The species lives in subarctic shrubland.
Subantarctic	0: The species does not live in subantarctic shrubland. 1: The species lives in subantarctic shrubland.
Boreal	0: The species does not live in boreal shrubland. 1: The species lives in boreal shrubland.
Temperate	0: The species does not live in temperate shrubland. 1: The species lives in temperate shrubland.
Subtropical/tropical dry	0: The species does not live in subtropical/tropical dry shrubland. 1: The species lives in subtropical/tropical dry shrubland.
Subtropical/tropical moist	0: The species does not live in subtropical/tropical moist shrubland. 1: The species lives in subtropical/tropical moist shrubland.
Tropical high altitude	0: The species does not live in tropical high altitude shrubland. 1: The species lives in tropical high altitude shrubland.
Mediterranean-type shrubby vegetation	0: The species does not live in mediterranean-type shrubby vegetation shrubland. 1: The species lives in mediterranean-type shrubby vegetation shrubland.
*Grassland*	
Tundra	0: The species does not live in tundra grassland. 1: The species lives in tropical tundra grassland.
Subarctic	0: The species does not live in subarctic grassland. 1: The species lives in tropical subarctic grassland.
Subantarctic	0: The species does not live in subantarctic grassland. 1: The species lives in tropical subantarctic grassland.
Temperate	0: The species does not live in temperate grassland. 1: The species lives in tropical temperate grassland.
Subtropical/tropical dry	0: The species does not live in subtropical/tropical dry grassland. 1: The species lives in tropical subtropical/tropical dry grassland.
Subtropical/tropical seasonally wet/ flooded	0: The species does not live in subtropical/tropical seasonally wet/ flooded grassland. 1: The species lives in tropical subtropical/tropical seasonally wet/ flooded grassland.
Subtropical/tropical high altitude	0: The species does not live in subtropical/ tropical high altitude grassland. 1: The species lives in tropical subtropical/ tropical high altitude grassland.
*Wetlands*	
Permanent rivers/streams/creeks (including waterfalls)	0: The species does not live in permanent rivers/streams/creeks (including waterfalls) wetlands. 1: The species lives in tropical permanent rivers/streams/creeks (including waterfalls) wetlands.
Intermittent/irregular rivers/streams/creeks	0: The species does not live in intermittent/irregular rivers/streams/creeks wetlands. 1: The species lives in tropical intermittent/irregular rivers/streams/creeks wetlands.
Shrub dominated wetlands	0: The species does not live in shrub dominated wetlands wetlands. 1: The species lives in tropical shrub dominated wetlands wetlands.
Bogs/marshes/swamps/fens/peatlands	0: The species does not live in bogs/marshes/swamps/fens/peatlands wetlands. 1: The species lives in tropical bogs/marshes/swamps/fens/peatlands wetlands.
Permanent freshwater lakes (>8ha)	0: The species does not live in permanent freshwater lakes (>8ha) wetlands. 1: The species lives in tropical permanent freshwater lakes (>8ha) wetlands.
Seasonal/intermittent freshwater lakes (>8ha)	0: The species does not live in seasonal/intermittent freshwater lakes (>8ha) wetlands. 1: The species lives in tropical seasonal/intermittent freshwater lakes (>8ha) wetlands.
Permanent freshwater marshes/pools (>8ha)	0: The species does not live in permanent freshwater marshes/pools (>8ha) wetlands. 1: The species lives in tropical permanent freshwater marshes/pools (>8ha) wetlands.
Seasonal/intermittent freshwater marshes/pools (<8ha)	0: The species does not live in seasonal/intermittent freshwater marshes/pools (<8ha) wetlands. 1: The species lives in tropical seasonal/intermittent freshwater marshes/pools (<8ha) wetlands.
Freshwater springs and oases	0: The species does not live in freshwater springs and oases wetlands. 1: The species lives in tropical freshwater springs and oases wetlands.
Tundra wetlands	0: The species does not live in tundra wetlands wetlands. 1: The species lives in tropical tundra wetlands wetlands.
Geothermal wetlands	0: The species does not live in geothermal wetlands wetlands. 1: The species lives in tropical geothermal wetlands wetlands.
Permanent inland deltas	0: The species does not live in permanent inland deltas wetlands. 1: The species lives in tropical permanent inland deltas wetlands.
Permanent saline, brackish or alkaline lakes	0: The species does not live in permanent saline, brackish or alkaline lakes wetlands. 1: The species lives in tropical permanent saline, brackish or alkaline lakes wetlands.
Seasonal/intermittent saline, brackish or alkaline lakes	0: The species does not live in seasonal/intermittent saline, brackish or alkaline lakes wetlands. 1: The species lives in tropical seasonal/intermittent saline, brackish or alkaline lakes wetlands.
Permanent saline, brackish or alkaline marshes/pools	0: The species does not live in permanent saline, brackish or alkaline marshes/pools wetlands. 1: The species lives in tropical permanent saline, brackish or alkaline marshes/pools wetlands.
Karst and other subterranean hydrological systems	0: The species does not live in karst and other subterranean hydrological systems wetlands. 1: The species lives in tropical karst and other subterranean hydrological systems wetlands.
*Rocky areas*	
Inland cliffs, moutain peaks	0: The species does not live in rock areas as inland cliffs, moutain peaks. 1: The species lives in rock areas as inland cliffs, moutain peaks.
*Caves and subterranean habitats (non-aquatic)*	
caves	0: The species does not live in caves and subterranean habitats. 1: The species lives in caves and subterranean habitats.
other subterranean habitats	0: The species does not live in other subterranean habitats. 1: The species lives in other subterranean habitats.
*Deserts*	
Hot	0: The species does not live in hot deserts. 1: The species lives in other hot deserts.
Temperate	0: The species does not live in temperate deserts. 1: The species lives in other temperate deserts.
Cold	0: The species does not live in cold deserts. 1: The species lives in other cold deserts.
*Artificial/terrestrial*	
Arable land	0: The species does not live in arable land. 1: The species lives in arable land.
Pastureland	0: The species does not live in pastureland. 1: The species lives in pastureland.
Plantations	0: The species does not live in plantations. 1: The species lives in plantations.
Rural gardens	0: The species does not live in rural gardens. 1: The species lives in rural gardens.
Urban areas	0: The species does not live in urban areas. 1: The species lives in urban areas.
Subtropical/tropical heavily degraded former forest	0: The species does not live in subtropical/tropical heavily degraded former forest. 1: The species lives in subtropical/tropical heavily degraded former forest.
*Other*	
Dunes	0: The species does not live in dunes. 1: The species lives in dunes.
**Topography**	
Modalities:	
Altitude min	Minimal attitude where the species was observed.
Altitude max	Maximal attitude where the species was observed.
**Biogeographical regions**	
Modalities:	
Alpine	0: The species does not live in the alpine biogeographical region. 1: The species lives in the alpine biogeographical region.
Anatolian	0: The species does not live in the anatolian biogeographical region. 1: The species lives in the anatolian biogeographical region.
Arctic	0: The species does not live in the arctic biogeographical region. 1: The species lives in the arctic biogeographical region.
Atlantic	0: The species does not live in the atlantic biogeographical region. 1: The species lives in the atlantic biogeographical region.
Black Sea	0: The species does not live in the black sea biogeographical region. 1: The species lives in the black sea biogeographical region.
Boreal	0: The species does not live in the boreal biogeographical region. 1: The species lives in the boreal biogeographical region.
Continental	0: The species does not live in the continentalbiogeographical region. 1: The species lives in the continental biogeographical region.
Mediterranean	0: The species does not live in the mediterranean biogeographical region. 1: The species lives in the mediterranean biogeographical region.
Macaronesian	0: The species does not live in the macaronesian biogeographical region. 1: The species lives in the macaronesian biogeographical region.
Pannonian	0: The species does not live in the pannonian biogeographical region. 1: The species lives in the pannonian biogeographical region.
Steppic	0: The species does not live in the steppic biogeographical region. 1: The species lives in the steppic biogeographical region.
**Distribution**	
Modalities:	
*Europe occupied UTM 50×50 km cells*	
Absolute	Spatial distribution of the species in Europe, calculated with GIS tools, in km².
*Asia*	Distribution of the species in Asia.
Absolute	Spatial distribution of the species in Asia, calculated with GIS tools, in km².
Punctual	0: The spatial distribution of the species is not rare. 1: The spatial distribution of the species is rare.
< 10%	0: Less than 10% of the spatial distribution of the species is not in Asia. 1: Less than 10% of the spatial distribution of the species is in Asia.
10–20%	0: Between 10 and 20% of the spatial distribution of the species are not in Asia. 1: Between 10 and 20% of the spatial distribution of the species are in Asia.
20–30%	0: Between 20 and 30% of the spatial distribution of the species are not in Asia. 1: Between 20 and 30% of the spatial distribution of the species are in Asia.
30–40%	0: Between 30 and 40% of the spatial distribution of the species are not in Asia. 1: Between 30 and 40% of the spatial distribution of the species are in Asia.
40–50%	0: Between 40 and 50% of the spatial distribution of the species are not in Asia. 1: Between 40 and 50% of the spatial distribution of the species are in Asia.
50–60%	0: Between 50 and 60% of the spatial distribution of the species are not in Asia. 1: Between 50 and 60% of the spatial distribution of the species are in Asia.
60–70%	0: Between 60 and 70% of the spatial distribution of the species are not in Asia. 1: Between 60 and 70% of the spatial distribution of the species are in Asia.
70–80%	0: Between 70 and 80% of the spatial distribution of the species are not in Asia. 1: Between 70 and 80% of the spatial distribution of the species are in Asia.
80–90%	0: Between 80 and 90% of the spatial distribution of the species are not in Asia. 1: Between 80 and 90% of the spatial distribution of the species are in Asia.
> 90%	0: Up to 90% of the spatial distribution of the species is not in Asia. 1: Up to 90% of the spatial distribution of the species is in Asia.
*Africa*	Distribution of the species in Africa.
Absolute	Spatial distribution of the species in Africa, calculated with GIS tools, in km².
Punctual	0: The spatial distribution of the species is not punctual. 1: The spatial distribution of the species is punctual.
<15%	0: Less than 15% of the spatial distribution of the species is not in Africa. 1: Less than 15% of the spatial distribution of the species is in Africa.
15–30%	0: Between 15 and 30% of the spatial distribution of the species are not in Africa. 1: Between 15 and 30% of the spatial distribution of the species are in Africa.
30–45%	0: Between 30 and 45% of the spatial distribution of the species are not in Africa. 1: Between 30 and 45% of the spatial distribution of the species are in Africa.
45–60%	0: Between 45 and 60% of the spatial distribution of the species are not in Africa. 1: Between 45 and 60% of the spatial distribution of the species are in Africa.
60–75%	0: Between 60 and 75% of the spatial distribution of the species are not in Africa. 1: Between 60 and 75% of the spatial distribution of the species are in Africa.
75–90%	0: Between 75 and 90% of the spatial distribution of the species are not in Africa. 1: Between 75 and 90% of the spatial distribution of the species are in Africa.
>90%	0: Up to 90% of the spatial distribution of the species is not in Africa. 1: Up to 90% of the spatial distribution of the species is in Africa.
*North America*	Distribution of the species in North America.
Absolute	Spatial distribution of the species in North America, calculated with GIS tools, in km².
Punctual	0: The spatial distribution of the species is not punctual. 1: The spatial distribution of the species is punctual.
<20%	0: Less than 20% of the spatial distribution of the species is not in North America. 1: Less than 20% of the spatial distribution of the species is in North America.
20–40%	0: Between 20 and 40% of the spatial distribution of the species are not in North America. 1: Between 20 and 40% of the spatial distribution of the species are in North America.
40–60%	0: Between 40 and 60% of the spatial distribution of the species are not in North America. 1: Between 40 and 60% of the spatial distribution of the species are in North America.
60–80%	0: Between 60 and 80% of the spatial distribution of the species are not in North America. 1: Between 60 and 80% of the spatial distribution of the species are in North America.
>80%	0: Up to 80% of the spatial distribution of the species is not in North America. 1: Up to 80% of the spatial distribution of the species is in North America.
*South America*	Distribution of the species in South America.
Absolute	Spatial distribution of the species in South America, calculated with GIS tools, in km².
Punctual	0: The spatial distribution of the species is not punctual. 1: The spatial distribution of the species is punctual.
<25%	0: Less than 25% of the spatial distribution of the species is not in South America. 1: Less than 25% of the spatial distribution of the species is in South America.
25–50%	0: Between 25 and 50% of the spatial distribution of the species are not in South America. 1: Between 25 and 50% of the spatial distribution of the species are in South America.
50–75%	0: Between 50 and 75% of the spatial distribution of the species are not in South America. 1: Between 50 and 75% of the spatial distribution of the species are in South America.
>75%	0: Up to 75% of the spatial distribution of the species is not in South America. 1: Up to 75% of the spatial distribution of the species is in South America.
*Australia*	Distribution of the species in Australia.
Absolute	Spatial distribution of the species in Australia, calculated with GIS tools, in km².
Punctual	0: The spatial distribution of the species is not punctual. 1: The spatial distribution of the species is punctual.
<50%	0: Less than 50% of the spatial distribution of the species is not in Australia. 1: Less than 50% of the spatial distribution of the species is in Australia.
>50%	0: Up to 50% of the spatial distribution of the species is not in Australia. 1: Up to 50% of the spatial distribution of the species is in Australia.
**Global Distribution**	
Modalities:	
Ubiquity	0: The species is not ubiquist. 1: The species is ubiquist.
Subendemic	0: The species is not subendemic. 1: The species is subendemic.
Endemic	0: The species is not endemic. 1: The species is endemic.

**Table 7.** Definition of the 3 traits related to threats in the European amphibian database. For all traits recorded, DD (data deficient) means that no data were reported in the literature.

**IUCN status**	IUCN threat status. Threatened species have one of the three following IUCN status: “vulnerable”, “endangered”, “critically endangered”.
Modalities:	
Data Deficient	0: Data sufficient for the species. 1: Data deficient for the species.
Least Concern	0: IUCN status for the species is not Least Concern. 1: IUCN status for the species is Least Concern.
Near Threatened	0: IUCN status for the species is not Near Threatened. 1: IUCN status for the species is Near Threatened.
Vulnerable	0: IUCN status for the species is not Vulnerable. 1: IUCN status for the species is Vulnerable.
Endangered	0: IUCN status for the species is not Endangered. 1: IUCN status for the species is Endangered.
Critically Endangered	0: IUCN status for the species is not Critically Endangered. 1: IUCN status for the species is Critically Endangered.
**Population trend**	Evolution of the population. Population trends vary between “decrease”, “stable” and “increase”.
Modalities:	
Data Deficient	0: Data sufficient for the species. 1: Data deficient for the species.
Decrease	0: Population trend for the species is not Decrease. 1: Population trend for the species is Decrease.
Stable	0: Population trend for the species is not Stable. 1: Population trend for the species is Stable.
Increase	0: Population trend for the species is not Increase. 1: Population trend for the species is Increase.
**Major Threats**	Major threats impacting the species.
Modalities:	
Habitat loss	0: Habitat loss does not affect the species. 1: Habitat loss affects the species.
Invasive alien	0: Invasive alien does not affect the species. 1: Invasive alien affects the species.
Harvesting	0: Harvesting does not affect the species. 1: Harvesting affects the species.
Accidental	0: Accidental does not affect the species. 1: Accidental affects the species.
Persecution	0: Persecution does not affect the species. 1: Persecution affects the species.
Pollution	0: Pollution does not affect the species. 1: Pollution affects the species.
Climate change	0: Climate change does not affect the species. 1: Climate change affects the species.
Natural disasters	0: Natural disasters do not affect the species. 1: Natural disasters affect the species.
Change dynamics	0: Change dynamics do not affect the species. 1: Change dynamics affect the species.
Intrinsic factors	0: Intrinsic factors do not affect the species. 1: Intrinsic factors affect the species.
Human disturbance	0: Human disturbance does not affect the species. 1: Human disturbance affects the species.
Unknown	0: Factors affecting the species are known. 1: Factors affecting the species are unknown.
